# Supplementary material for: Integrating Parental Phenotypic Data Enhances Prediction Accuracy of Hybrids in Wheat Traits
Source: Genes (Basel). 2023 Feb 2;14(2):395. doi: 10.3390/genes14020395 (PMC9957193; doi:10.3390/genes14020395)
Supplement: Supplementary file 1 [file genes-14-00395-s001.zip › genes-2132017-supplementary.pdf]

Table S1. Prediction performance for each trait in each environment and across environments for each trait (global) for type A models (MA, MA_C and MA_AC) in terms of mean squared error (MSE) under untested lines in tested environments across validation strategy. The Relative efficiency (RE_MSE) were computed dividing the MSE of model MA by the MSE of model MA_C, also was computed the RE_MSE for comparing models MA vs MA_AC and for comparing models MA_C vs MA_AC.

Model	Predictor	Trait	Year	MSE	MSE_SE	RE_MSE	Method
MA	E+M+F+H+ME+FE+HE	GY	1	0.287	0.023	-	-
MA	E+M+F+H+ME+FE+HE	GY	2	0.223	0.015	-	-
MA	E+M+F+H+ME+FE+HE	GY	3	0.213	0.021	-	-
MA	E+M+F+H+ME+FE+HE	GY	Global	0.200	0.015	-	-
MA	E+M+F+H+ME+FE+HE	DTH	1	2.946	0.394	-	-
MA	E+M+F+H+ME+FE+HE	DTH	2	2.224	0.168	-	-
MA	E+M+F+H+ME+FE+HE	DTH	3	3.963	0.321	-	-
MA	E+M+F+H+ME+FE+HE	DTH	Global	3.058	0.186	-	-
MA	E+M+F+H+ME+FE+HE	DTF	1	2.904	0.366	-	-
MA	E+M+F+H+ME+FE+HE	DTF	2	2.168	0.165	-	-
MA	E+M+F+H+ME+FE+HE	DTF	3	3.931	0.323	-	-
MA	E+M+F+H+ME+FE+HE	DTF	Global	3.010	0.172	-	-
MA_C	E+M+F+H+ME+FE+HE+X_C	GY	1	0.253	0.020	1.135	MA vs MA_C
MA_C	E+M+F+H+ME+FE+HE+X_C	GY	2	0.231	0.022	0.962	MA vs MA_C
MA_C	E+M+F+H+ME+FE+HE+X_C	GY	3	0.180	0.009	1.179	MA vs MA_C
MA_C	E+M+F+H+ME+FE+HE+X_C	GY	Global	0.175	0.010	1.141	MA vs MA_C
MA_C	E+M+F+H+ME+FE+HE+X_C	DTH	1	2.872	0.317	1.026	MA vs MA_C
MA_C	E+M+F+H+ME+FE+HE+X_C	DTH	2	1.949	0.117	1.141	MA vs MA_C

MA_C	E+M+F+H+ME+FE+HE+X_C	DTH	3	3.280	0.228	1.208	MA vs MA_C
MA_C	E+M+F+H+ME+FE+HE+X_C	DTH	Global	2.601	0.113	1.176	MA vs MA_C
MA_C	E+M+F+H+ME+FE+HE+X_C	DTF	1	2.849	0.289	1.019	MA vs MA_C
MA_C	E+M+F+H+ME+FE+HE+X_C	DTF	2	1.924	0.081	1.126	MA vs MA_C
MA_C	E+M+F+H+ME+FE+HE+X_C	DTF	3	3.255	0.246	1.208	MA vs MA_C
MA_C	E+M+F+H+ME+FE+HE+X_C	DTF	Global	2.575	0.117	1.169	MA vs MA_C
MA_AC	E+M+F+H+ME+FE+HE+X_AC	GY	1	0.253	0.020	1.136	MA vs MA_AC
MA_AC	E+M+F+H+ME+FE+HE+X_AC	GY	2	0.230	0.023	0.969	MA vs MA_AC
MA_AC	E+M+F+H+ME+FE+HE+X_AC	GY	3	0.183	0.008	1.162	MA vs MA_AC
MA_AC	E+M+F+H+ME+FE+HE+X_AC	GY	Global	0.176	0.010	1.137	MA vs MA_AC
MA_AC	E+M+F+H+ME+FE+HE+X_AC	DTH	1	2.821	0.314	1.044	MA vs MA_AC
MA_AC	E+M+F+H+ME+FE+HE+X_AC	DTH	2	1.932	0.122	1.151	MA vs MA_AC
MA_AC	E+M+F+H+ME+FE+HE+X_AC	DTH	3	3.283	0.249	1.207	MA vs MA_AC
MA_AC	E+M+F+H+ME+FE+HE+X_AC	DTH	Global	2.597	0.115	1.178	MA vs MA_AC
MA_AC	E+M+F+H+ME+FE+HE+X_AC	DTF	1	2.803	0.287	1.036	MA vs MA_AC
MA_AC	E+M+F+H+ME+FE+HE+X_AC	DTF	2	1.909	0.081	1.136	MA vs MA_AC
MA_AC	E+M+F+H+ME+FE+HE+X_AC	DTF	3	3.284	0.260	1.197	MA vs MA_AC
MA_AC	E+M+F+H+ME+FE+HE+X_AC	DTF	Global	2.579	0.119	1.167	MA vs MA_AC
MA_AC	E+M+F+H+ME+FE+HE+X_AC	GY	1	-	-	1.001	MA_C vs MA_AC
MA_AC	E+M+F+H+ME+FE+HE+X_AC	GY	2	-	-	1.007	MA_C vs MA_AC
MA_AC	E+M+F+H+ME+FE+HE+X_AC	GY	3	-	-	0.986	MA_C vs MA_AC

MA_AC	E+M+F+H+ME+ FE+HE+X_AC	GY	Global	-	-	0.996	MA_C vs MA_AC
MA_AC	E+M+F+H+ME+ FE+HE+X_AC	DTH	1	-	-	1.018	MA_C vs MA_AC
MA_AC	E+M+F+H+ME+ FE+HE+X_AC	DTH	2	-	-	1.009	MA_C vs MA_AC
MA_AC	E+M+F+H+ME+ FE+HE+X_AC	DTH	3	-	-	0.999	MA C vs MA_AC
MA_AC	E+M+F+H+ME+ FE+HE+X_AC	DTH	Global	-	-	1.002	MA_C vs MA_AC
MA_AC	E+M+F+H+ME+ FE+HE+X_AC	DTF	1	-	-	1.016	MA_C vs MA_AC
MA_AC	E+M+F+H+ME+ FE+HE+X_AC	DTF	2	-	-	1.008	MA_C vs MA_AC
MA_AC	E+M+F+H+ME+ FE+HE+X_AC	DTF	3	-	-	0.991	MA_C vs MA_AC
MA_AC	E+M+F+H+ME+ FE+HE+X_AC	DTF	Global	-	-	0.998	MA_C vs MA_AC

**Table S2.** Prediction performance for each trait in each environment and across environments for each trait (global) for type B models (MB, MB_C and MB_AC) in terms of mean squared error (MSE) under untested lines in tested environments across validation strategy. The Relative efficiency (RE_MSE) were computed dividing the MSE of model MB by the MSE of model MB_C, also was computed the RE_MSE for comparing models MB vs MB_AC and for comparing models MB_C vs MB_AC.							
**Model**	**Predictor**	**Trait**	**Year**	**MSE**	**MSE_SE**	**RE_MSE**	**Method**
MB	E+M+F+H+ME+FE+HE	GY	1	0.267	0.018	-	-
MB	E+M+F+H+ME+FE+HE	GY	2	0.215	0.015	-	-
MB	E+M+F+H+ME+FE+HE	GY	3	0.189	0.013	-	-
MB	E+M+F+H+ME+FE+HE	GY	Global	0.178	0.008	-	-
MB	E+M+F+H+ME+FE+HE	DTH	1	3.006	0.346	-	-
MB	E+M+F+H+ME+FE+HE	DTH	2	2.030	0.102	-	-
MB	E+M+F+H+ME+FE+HE	DTH	3	3.820	0.240	-	-
MB	E+M+F+H+ME+FE+HE	DTH	Global	2.946	0.131	-	-
MB	E+M+F+H+ME+FE+HE	DTF	1	2.986	0.327	-	-
MB	E+M+F+H+ME+FE+HE	DTF	2	2.055	0.107	-	-
MB	E+M+F+H+ME+FE+HE	DTF	3	3.759	0.250	-	-
MB	E+M+F+H+ME+FE+HE	DTF	Global	2.906	0.123	-	-
MB_C	E+M+F+H+ME+FE+HE+X_C	GY	1	0.254	0.022	1.050	MB vs MB_C
MB_C	E+M+F+H+ME+FE+HE+X_C	GY	2	0.225	0.020	0.958	MB vs MB_C
MB_C	E+M+F+H+ME+FE+HE+X_C	GY	3	0.170	0.007	1.114	MB vs MB_C
MB_C	E+M+F+H+ME+FE+HE+X_C	GY	Global	0.168	0.009	1.055	MB vs MB_C
MB_C	E+M+F+H+ME+FE+HE+X_C	DTH	1	3.007	0.269	1.000	MB vs MB_C
MB_C	E+M+F+H+ME+FE+HE+X_C	DTH	2	1.863	0.086	1.089	MB vs MB_C
MB_C	E+M+F+H+ME+FE+HE+X_C	DTH	3	3.235	0.208	1.181	MB vs MB_C
MB_C	E+M+F+H+ME+FE+HE+X_C	DTH	Global	2.630	0.120	1.120	MB vs MB_C
MB_C	E+M+F+H+ME+FE+HE+X_C	DTF	1	2.981	0.248	1.002	MB vs MB_C

MB_C	E+M+F+H+ME+FE+HE+X_C	DTF	2	1.896	0.038	1.084	MB vs MB_C
MB_C	E+M+F+H+ME+FE+HE+X_C	DTF	3	3.185	0.214	1.180	MB vs MB_C
MB_C	E+M+F+H+ME+FE+HE+X_C	DTF	Global	2.596	0.112	1.119	MB vs MB_C
MB_AC	E+M+F+H+ME+FE+HE+X_AC	GY	1	0.254	0.021	1.052	MB vs MB_AC
MB_AC	E+M+F+H+ME+FE+HE+X_AC	GY	2	0.221	0.020	0.973	MB vs MB_AC
MB_AC	E+M+F+H+ME+FE+HE+X_AC	GY	3	0.172	0.007	1.101	MB vs MB_AC
MB_AC	E+M+F+H+ME+FE+HE+X_AC	GY	Global	0.169	0.009	1.053	MB vs MB_AC
MB_AC	E+M+F+H+ME+FE+HE+X_AC	DTH	1	2.872	0.258	1.047	MB vs MB_AC
MB_AC	E+M+F+H+ME+FE+HE+X_AC	DTH	2	1.865	0.091	1.088	MB vs MB_AC
MB_AC	E+M+F+H+ME+FE+HE+X_AC	DTH	3	3.221	0.203	1.186	MB vs MB_AC
MB_AC	E+M+F+H+ME+FE+HE+X_AC	DTH	Global	2.601	0.112	1.133	MB vs MB_AC
MB_AC	E+M+F+H+ME+FE+HE+X_AC	DTF	1	2.864	0.239	1.042	MB vs MB_AC
MB_AC	E+M+F+H+ME+FE+HE+X_AC	DTF	2	1.882	0.034	1.092	MB vs MB_AC
MB_AC	E+M+F+H+ME+FE+HE+X_AC	DTF	3	3.185	0.213	1.180	MB vs MB_AC
MB_AC	E+M+F+H+ME+FE+HE+X_AC	DTF	Global	2.573	0.110	1.129	MB vs MB_AC
MB_AC	E+M+F+H+ME+FE+HE+X_AC	GY	1	-	-	1.002	MB C vs MB_AC
MB_AC	E+M+F+H+ME+FE+HE+X_AC	GY	2	-	-	1.016	MB C vs MB_AC
MB_AC	E+M+F+H+ME+FE+HE+X_AC	GY	3	-	-	0.988	MB C vs MB_AC
MB_AC	E+M+F+H+ME+FE+HE+X_AC	GY	Global	-	-	0.998	MB C vs MB_AC
MB_AC	E+M+F+H+ME+FE+HE+X_AC	DTH	1	-	-	1.047	MB C vs MB_AC

MB_AC	E+M+F+H+ME+FE+HE+X_AC	DTH	2	-	-	0.999	MB C vs MB_AC
MB_AC	E+M+F+H+ME+FE+HE+X_AC	DTH	3	-	-	1.004	MB C vs MB_AC
MB_AC	E+M+F+H+ME+FE+HE+X_AC	DTH	Global	-	-	1.011	MB C vs MB_AC
MB_AC	E+M+F+H+ME+FE+HE+X_AC	DTF	1	-	-	1.041	MB C vs MB_AC
MB_AC	E+M+F+H+ME+FE+HE+X_AC	DTF	2	-	-	1.008	MB C vs MB_AC
MB_AC	E+M+F+H+ME+FE+HE+X_AC	DTF	3	-	-	1.000	MB C vs MB_AC
MB_AC	E+M+F+H+ME+FE+HE+X_AC	DTF	Global	-	-	1.009	MB C vs MB_AC

**Table S3.** Prediction performance across environments for each trait (global) for all models MA, MA_C, MA_AC, MB, MB_C, MB_AC, MC, MC_C, MC_AC, MD, MD_C and MD_AC in terms of mean squared error (MSE) under untested lines in tested environments across validation strategy. The Relative efficiency (RE_MSE) were computed dividing the MSE of model MX by the MSE of model MX_C or MX_AC model, with X taking the values of A, B, C and D.						
**Model**	**Predictor**	**Trait**	**MSE**	**MSE_SE**	**RE_MSE**	**Method**
MA	E+M+F+H+ME+FE+HE	GY	0.200	0.015	-	-
MA	E+M+F+H+ME+FE+HE	DTH	3.058	0.186	-	-
MA	E+M+F+H+ME+FE+HE	DTF	3.010	0.172	-	-
MB	E+M+F+H+ME+FE+HE	GY	0.178	0.008	-	-
MB	E+M+F+H+ME+FE+HE	DTH	2.946	0.131	-	-
MB	E+M+F+H+ME+FE+HE	DTF	2.906	0.123	-	-
MC	E+H+HE	GY	0.342	0.018	-	-
MC	E+H+HE	DTH	9.208	0.385	-	-
MC	E+H+HE	DTF	8.819	0.344	-	-
MD	E+H+HE	GY	0.179	0.009	-	-
MD	E+H+HE	DTH	2.999	0.122	-	-
MD	E+H+HE	DTF	2.952	0.122	-	-
MA_C	E+M+F+H+ME+FE+HE+X_C	GY	0.175	0.010	1.141	MA vs MA_C
MA_C	E+M+F+H+ME+FE+HE+X_C	DTH	2.601	0.113	1.176	MA vs MA_C
MA_C	E+M+F+H+ME+FE+HE+X_C	DTF	2.575	0.117	1.169	MA vs MB_C
MB_C	E+M+F+H+ME+FE+HE+X_C	GY	0.168	0.009	1.055	MB vs MB_C
MB_C	E+M+F+H+ME+FE+HE+X_C	DTH	2.630	0.120	1.120	MB vs MB_C
MB_C	E+M+F+H+ME+FE+HE+X_C	DTF	2.596	0.112	1.119	MB vs MB_C
MC_C	E+H+HE+X_C	GY	0.226	0.010	1.514	MC vs MC_C
MC_C	E+H+HE+X_C	DTH	4.710	0.232	1.955	MC vs MC_C

MC_C	E+H+HE+X_C	DTF	4.490	0.228	1.964	MC vs MC_C
MD_C	E+H+HE+X_C	GY	0.169	0.009	1.064	MD vs MD_C
MD_C	E+H+HE+X_C	DTH	2.697	0.102	1.112	MD vs MD_C
MD_C	E+H+HE+X_C	DTF	2.669	0.100	1.106	MD vs MD_C
MA_AC	E+M+F+H+ME+FE+HE+X_AC	GY	0.176	0.010	1.137	MA vs MA_AC
MA_AC	E+M+F+H+ME+FE+HE+X_AC	DTH	2.597	0.115	1.178	MA vs MA_AC
MA_AC	E+M+F+H+ME+FE+HE+X_AC	DTF	2.579	0.119	1.167	MA vs MA_AC
MB_AC	E+M+F+H+ME+FE+HE+X_AC	GY	0.169	0.009	1.053	MB vs MB_AC
MB_AC	E+M+F+H+ME+FE+HE+X_AC	DTH	2.601	0.112	1.133	MB vs MB_AC
MB_AC	E+M+F+H+ME+FE+HE+X_AC	DTF	2.573	0.110	1.129	MB vs MB_AC
MC_AC	E+H+HE+X_AC	GY	0.221	0.011	1.551	MC vs MC_AC
MC_AC	E+H+HE+X_AC	DTH	4.592	0.258	2.005	MC vs MC_AC
MC_AC	E+H+HE+X_AC	DTF	4.418	0.239	1.996	MC vs MC_AC
MD_AC	E+H+HE+X_AC	GY	0.169	0.009	1.060	MD vs MD_AC
MD_AC	E+H+HE+X_AC	DTH	2.678	0.100	1.120	MD vs MD_AC
MD_AC	E+H+HE+X_AC	DTF	2.651	0.099	1.113	MD vs MD_AC

Table S4. Prediction performance for each trait in each environment and across environments for each trait (global) for type C models (MC, MC_C and MC_AC) in terms of mean squared error (MSE) under untested lines in tested environments across validation strategy. The Relative efficiency (RE_MSE) were computed dividing the MSE of model MC by the MSE of model MC_C, also was computed the RE_MSE for comparing models MC vs MC_AC and for comparing models MC_C vs MC_AC.

Model	Predictor	Trait	Year	MSE	MSE_SE	RE_MSE	Method
MC	E+H+HE	GY	1	0.396	0.031	-	-
MC	E+H+HE	GY	2	0.339	0.009	-	-
MC	E+H+HE	GY	3	0.366	0.028	-	-
MC	E+H+HE	GY	Global	0.342	0.018	-	-
MC	E+H+HE	DTH	1	7.099	0.504	-	-
MC	E+H+HE	DTH	2	5.940	0.224	-	-
MC	E+H+HE	DTH	3	11.120	0.663	-	-
MC	E+H+HE	DTH	Global	9.208	0.385	-	-
MC	E+H+HE	DTF	1	6.823	0.494	-	-
MC	E+H+HE	DTF	2	5.466	0.219	-	-
MC	E+H+HE	DTF	3	10.806	0.630	-	-
MC	E+H+HE	DTF	Global	8.819	0.344	-	-
MC_C	E+H+HE+X_C	GY	1	0.282	0.013	1.401	MC vs MC_C
MC_C	E+H+HE+X_C	GY	2	0.294	0.022	1.151	MC vs MC_C
MC_C	E+H+HE+X_C	GY	3	0.242	0.008	1.510	MC vs MC_C
MC_C	E+H+HE+X_C	GY	Global	0.226	0.010	1.514	MC vs MC_C
MC_C	E+H+HE+X_C	DTH	1	4.858	0.446	1.461	MC vs MC_C
MC_C	E+H+HE+X_C	DTH	2	2.990	0.158	1.986	MC vs MC_C
MC_C	E+H+HE+X_C	DTH	3	5.590	0.314	1.989	MC vs MC_C
MC_C	E+H+HE+X_C	DTH	Global	4.710	0.232	1.955	MC vs MC_C

MC_C	E+H+HE+X_C	DTF	1	4.584	0.392	1.488	MC vs MC_C
MC_C	E+H+HE+X_C	DTF	2	2.795	0.108	1.955	MC vs MC_C
MC_C	E+H+HE+X_C	DTF	3	5.450	0.334	1.983	MC vs MC_C
MC_C	E+H+HE+X_C	DTF	Global	4.490	0.228	1.964	MC vs MC_C
MC_AC	E+H+HE+X_AC	GY	1	0.276	0.013	1.433	MC vs MC_AC
MC_AC	E+H+HE+X_AC	GY	2	0.278	0.022	1.218	MC vs MC_AC
MC_AC	E+H+HE+X_AC	GY	3	0.242	0.009	1.511	MC vs MC_AC
MC_AC	E+H+HE+X_AC	GY	Global	0.221	0.011	1.551	MC vs MC_AC
MC_AC	E+H+HE+X_AC	DTH	1	4.567	0.416	1.554	MC vs MC_AC
MC_AC	E+H+HE+X_AC	DTH	2	2.940	0.147	2.021	MC vs MC_AC
MC_AC	E+H+HE+X_AC	DTH	3	5.520	0.350	2.014	MC vs MC_AC
MC_AC	E+H+HE+X_AC	DTH	Global	4.592	0.258	2.005	MC vs MC_AC
MC_AC	E+H+HE+X_AC	DTF	1	4.377	0.377	1.559	MC vs MC_AC
MC_AC	E+H+HE+X_AC	DTF	2	2.771	0.101	1.973	MC vs MC_AC
MC_AC	E+H+HE+X_AC	DTF	3	5.418	0.350	1.994	MC vs MC_AC
MC_AC	E+H+HE+X_AC	DTF	Global	4.418	**0.239**	**1.996**	MC vs MC_AC
MC_AC	E+H+HE+X_AC	GY	1	-	-	1.022	MC C vs MC_AC
MC_AC	E+H+HE+X_AC	GY	2	-	-	1.059	MC C vs MC_AC

MC_AC	E+H+HE+X_AC	GY	3	-	-	1.000	MC C vs MC_AC
MC_AC	E+H+HE+X_AC	GY	Global	-	-	1.024	MC C vs MC_AC
MC_AC	E+H+HE+X_AC	DTH	1	-	-	1.064	MC C vs MC_AC
MC_AC	E+H+HE+X_AC	DTH	2	-	-	1.017	MC C vs MC_AC
MC_AC	E+H+HE+X_AC	DTH	3	-	-	1.013	MC C vs MC_AC
MC_AC	E+H+HE+X_AC	DTH	Global	-	-	1.026	MC C vs MC_AC
MC_AC	E+H+HE+X_AC	DTF	1	-	-	1.047	MC C vs MC_AC
MC_AC	E+H+HE+X_AC	DTF	2	-	-	1.009	MC C vs MC_AC
MC_AC	E+H+HE+X_AC	DTF	3	-	-	1.006	MC C vs MC_AC
MC_AC	E+H+HE+X_AC	DTF	Global	-	-	1.016	MC C vs MC_AC

Table S5. Prediction performance for each trait in each environment and across environments for each trait (global) for type D models (MD, MD_C and MD_AC) in terms of mean squared error (MSE) under untested lines in tested environments across validation strategy. The Relative efficiency (RE_MSE) were computed dividing the MSE of model MD by the MSE of model MD_C, also was computed the RE_MSE for comparing models MD vs MD_AC and for comparing models MD_C vs MD_AC.

Model	Predictor	Trait	Year	MSE	MSE_SE	RE_ MSE	Method
MD	E+H+HE	GY	1	0.268	0.019	-	-
MD	E+H+HE	GY	2	0.214	0.015	-	-
MD	E+H+HE	GY	3	0.192	0.013	-	-
MD	E+H+HE	GY	Global	0.179	0.009	-	-
MD	E+H+HE	DTH	1	2.990	0.339	-	-
MD	E+H+HE	DTH	2	2.062	0.093	-	-
MD	E+H+HE	DTH	3	3.923	0.286	-	-
MD	E+H+HE	DTH	Global	2.999	0.122	-	-
MD	E+H+HE	DTF	1	2.964	0.318	-	-
MD	E+H+HE	DTF	2	2.089	0.090	-	-
MD	E+H+HE	DTF	3	3.854	0.295	-	-
MD	E+H+HE	DTF	Global	2.952	0.122	-	-
MD_C	E+H+HE+X_C	GY	1	0.255	0.022	1.052	MD vs MD_C
MD_C	E+H+HE+X_C	GY	2	0.224	0.020	0.954	MD vs MD_C
MD_C	E+H+HE+X_C	GY	3	0.170	0.008	1.129	MD vs MD_C
MD_C	E+H+HE+X_C	GY	Global	0.169	0.009	1.064	MD vs MD_C
MD_C	E+H+HE+X_C	DTH	1	3.029	0.268	0.987	MD vs MD_C
MD_C	E+H+HE+X_C	DTH	2	1.904	0.099	1.083	MD vs MD_C
MD_C	E+H+HE+X_C	DTH	3	3.339	0.224	1.175	MD vs MD_C
MD_C	E+H+HE+X_C	DTH	Global	2.697	0.102	1.112	MD vs MD_C

MD_C	E+H+HE+X_C	DTF	1	3.006	0.243	0.986	MD vs MD_C
MD_C	E+H+HE+X_C	DTF	2	1.937	0.049	1.079	MD vs MD_C
MD_C	E+H+HE+X_C	DTF	3	3.297	0.228	1.169	MD vs MD_C
MD_C	E+H+HE+X_C	DTF	Global	2.669	0.100	1.106	MD vs MD_C
MD_AC	E+H+HE+X_AC	GY	1	0.255	0.021	1.053	MD vs MD_AC
MD_AC	E+H+HE+X_AC	GY	2	0.222	0.020	0.965	MD vs MD_AC
MD_AC	E+H+HE+X_AC	GY	3	0.173	0.008	1.113	MD vs MD_AC
MD_AC	E+H+HE+X_AC	GY	Global	0.169	0.009	1.060	MD vs MD_AC
MD_AC	E+H+HE+X_AC	DTH	1	2.905	0.263	1.029	MD vs MD_AC
MD_AC	E+H+HE+X_AC	DTH	2	1.910	0.104	1.080	MD vs MD_AC
MD_AC	E+H+HE+X_AC	DTH	3	3.341	0.221	1.174	MD vs MD_AC
MD_AC	E+H+HE+X_AC	DTH	Global	2.678	0.100	1.120	MD vs MD_AC
MD_AC	E+H+HE+X_AC	DTF	1	2.905	0.241	1.020	MD vs MD_AC
MD_AC	E+H+HE+X_AC	DTF	2	1.922	0.042	1.087	MD vs MD_AC
MD_AC	E+H+HE+X_AC	DTF	3	3.305	0.226	1.166	MD vs MD_AC
MD_AC	E+H+HE+X_AC	DTF	Global	2.651	0.099	1.113	MD vs MD_AC
MD_AC	E+H+HE+X_AC	GY	1	-	-	1.002	MD C vs MD_AC
MD_AC	E+H+HE+X_AC	GY	2	-	-	1.012	MD C vs MD_AC
MD_AC	E+H+HE+X_AC	GY	3	-	-	0.986	MD C vs MD_AC
MD_AC	E+H+HE+X_AC	GY	Global	-	-	0.996	MD C vs MD_AC

MD_AC	E+H+HE+X_AC	DTH	1	-	-	1.043	MD C vs MD_AC
MD_AC	E+H+HE+X_AC	DTH	2	-	-	0.997	MD C vs MD_AC
MD_AC	E+H+HE+X_AC	DTH	3	-	-	0.999	MD C vs MD_AC
MD_AC	E+H+HE+X_AC	DTH	Global	-	-	1.007	MD C vs MD_AC
MD_AC	E+H+HE+X_AC	DTF	1	-	-	1.035	MD C vs MD_AC
MD_AC	E+H+HE+X_AC	DTF	2	-	-	1.008	MD C vs MD_AC
MD_AC	E+H+HE+X_AC	DTF	3	-	-	0.998	MD C vs MD_AC
